# Association Between a *TLR2* Gene Polymorphism (rs3804099) and Proteinuria in Kidney Transplantation Recipients

**DOI:** 10.3389/fgene.2021.798001

**Published:** 2022-02-21

**Authors:** Shuang Fei, Zeping Gui, Dengyuan Feng, Zijie Wang, Ming Zheng, Hao Chen, Li Sun, Jun Tao, Zhijian Han, Xiaobing Ju, Min Gu, Ruoyun Tan, Xinli Li

**Affiliations:** ^1^ Department of Urology, The First Affiliated Hospital with Nanjing Medical University, Nanjing, China; ^2^ Department of Urology, The Second Affiliated Hospital with Nanjing Medical University, Nanjing, China; ^3^ Department of Cardiology, The First Affiliated Hospital with Nanjing Medical University, Nanjing, China

**Keywords:** kidney transplantation, proteinuria, single-nucleotide polymorphisms, high-throughput sequencing, Toll-like receptor 2

## Abstract

**Background:** The occurrence of proteinuria is one of the evaluation indicators of transplanted kidney damage and becomes an independent risk factor for poor prognosis after kidney transplantation. Our research sought to understand these potential associations and detect the underlying impact of single-nucleotide polymorphisms (SNPs) on proteinuria in kidney transplant recipients.

**Materials and Methods:** There were 200 recipients enrolled in this study, from which blood samples were extracted for SNP mutation–related gene detection. RNA sequencing was performed in kidney tissues after kidney transplantation, and the significantly differentially expressed genes (DEGs) were analyzed between the control group and the proteinuria group. Then, the intersection of genes with SNP mutations and DEGs was conducted to obtain the target genes. Multiple genetic models were used to investigate the relationship between SNPs and proteinuria. In addition, the effect of SNP mutation in the target gene was further validated in human renal podocytes.

**Results:** According to the sequencing results, 26 significant SNP mutated genes and 532 DEGs were found associated with proteinuria after kidney transplantation. The intersection of SNP mutated genes and DEGs showed that the Toll-like receptor 2 (TLR2) gene was significantly increased in the transplanted renal tissues of patients with proteinuria after kidney transplantation, which was consistent with the results of immunohistochemical staining. Further inheritance model results confirmed that mutations at rs3804099 of the TLR2 gene had significant influence on the occurrence of proteinuria after kidney transplantation. In the *in vitro* validation, we found that, after the mutation of rs3804099 on the TLR2 gene, the protein expressions of podocalyxin and nephrin in podocytes were significantly decreased, while the protein expressions of desmin and apoptosis markers were significantly increased. The results of flow cytometry also showed that the mutation of rs3804099 on the TLR2 gene significantly increased the apoptotic rate of podocytes.

**Conclusion:** Our study suggested that the mutation of rs3804099 on the TLR2 gene was significantly related to the generation of proteinuria after kidney transplantation. Our data provide insights into the prediction of proteinuria and may imply potential individualized therapy for patients after kidney transplantation.

## Background

With the improvement of organ transplantation methods and the application of new immunosuppressive agents, the short-term survival rate of transplanted kidney has been significantly ameliorated. However, the long-term survival rate of transplanted kidney needs to be improved, and the long-term complications after kidney transplantation are receiving more attention. Consistent with proteinuria in common chronic kidney diseases, persistent proteinuria after kidney transplantation is closely connected with the prognosis of kidney transplantation (Blasius and Beutler, 2010). The incidence of proteinuria 1 year after kidney transplantation is 15–20%. The long-term survival of transplanted kidney is directly related to the occurrence of proteinuria, which is an independent risk factor affecting allograft survival and leads to the death of patients after transplantation (Fernandez-Fresnedo et al., 2004). Immune and non-immune factors after kidney transplantation contribute to proteinuria (Sancho et al., 2007). Studies have shown that enhanced immunosuppressive therapy is ineffective for post-transplantation proteinuria. The generation of proteinuria after kidney transplantation attributes to chronic rejection, chronic graft nephropathy, glomerulonephritis after kidney transplantation, acute rejection, and cyclosporine nephrotoxicity (Reichel et al., 2004). Effective control of proteinuria after transplantation can reduce the damage to the transplanted kidney.

Nowadays, abundant research studies have demonstrated that gene polymorphisms are associated with kidney disease. The mutation and polymorphism of nephrin gene NPHS1 impact the development of congenital nephrotic syndrome of Finnish type (CNF), MCNS, and other diseases as well as the occurrence of its proteinuria. Mutations in the NPHS2 gene of podocin contribute to steroid-resistant nephrotic syndrome (SRNS), most of which are familial but may also are sporadic (Rood et al., 2019). Mutations in the podocin gene are also relevant to non-diabetic end-stage renal disease in African Americans (Menara et al., 2020). And clear correlation between the polymorphism of this gene and the occurrence of proteinuria has been validated in the general population. This evidence all indicates that genetic mutations obviously have an influence on the pathogenesis of kidney disease.

Toll-like receptors (TLRs), a family of structural recognition receptors across cell membranes, play an important role in inflammation, immune response, and tumorigenesis, where TLR2 is a crucial member. The coding gene of TLR2 is located on the 4q32 region of chromosome 4, and TLR2 is mainly expressed on the surface of peripheral blood leukocytes. TLR2 can interact with bacteria and endogenous ligands to activate transcription factors, such as NF-κB and AP-1 (Harding and Boom, 2010; Vilahur and Badimon, 2014). Current studies have validated that, after tissue ischemic injury, numerous apoptotic cells could emerge in the organs and generate a series of immune responses such as host defense and injury repair responses (Marshak-Rothstein, 2006; Obhrai and Goldstein, 2006; Seki and Brenner, 2008). The accumulation of inflammatory cells along with the release of inflammatory factors can damage the tissues. The loss of TLR2 can lead to kidney damage, leukocyte influx, and renal tubular damage during renal ischemia–reperfusion. Ding et al. (2015) reported that proteinuria might exhibit an endogenous danger-associated molecular pattern (DAMP) that induced tubulointerstitial inflammation *via* TLR2–MyD88–NF-κB pathway activation. In this study, the correlation between TLR2-regulated gene expressions and proteinuria after kidney transplantation will be investigated.

## Materials and Methods

### Ethics Statement

All research design, patient registration, and procedure agreement were approved by the local ethics committee of the First Affiliated Hospital of Nanjing Medical University (2016-SR-029). All kidney-transplanted recipients expressed their knowledge and understanding of the experimental process and provided written informed consent at the same time. All our research processes follow the ethical standards of the “Helsinki Declaration” and the “Istanbul Declaration.”

### Study Design and Population

We use a single-center, retrospective, cohort study method to explore the impact of single-nucleotide polymorphisms of genes based on TLR2-related signaling pathways and the progress of proteinuria in kidney-transplanted recipients. A total of 200 recipients who received kidney transplanted in the Kidney Transplant Center at the First Affiliated Hospital of Nanjing Medical University from February 1, 2015, to September 1, 2018, participated in this study. There is no significant transplanted renal function failure or decline during the current follow-up. The detailed research methods including the inclusion and exclusion criteria were described in detail in our previous studies (Wang et al., 2018). The clinical data, including age, gender, height, independence, and immunosuppressive protocol, were recorded by one of the authors, Zeping Gui.

### Immunosuppressive Protocols

Our center selected triple immunosuppressive regimen [cyclosporin A or tacrolimus, combined with mycophenolate mofetil (MMF) and prednisone] or quadruple immunosuppressive regimen (cyclosporin A or tacrolimus, prednisone, MMF combined with sirolimus) as maintenance-period immunosuppressant treatment. We adjusted the dosage of the corresponding immunosuppressive regimen according to drug concentration levels and blood creatinine level. Our previous research studies had reported corresponding details about detailed information and methodologies for immunosuppressive agent schedules (Wang et al., 2018).

### Sample Collection and Next-Generation Sequencing (NGS)

Peripheral blood (2 ml) of each patient was used for DNA extraction. We quantitatively analyzed the concentration and purity of genomic DNA (gDNA) and assessed the integrity of genes by agarose gel electrophoresis. We chose target-specific target regions from a random pool containing upstream and downstream oligonucleotides and gDNA hybrids. Then, we dispersed gDNA and selectively amplified adapter-ligated DNA, limited-cycle polymerase chain reaction (PCR). We denatured the captured library and loaded it into an Illumina cBot instrument manufacturer’s protocol. Subsequently, we analyzed the sequencing data based on the human available data reference sequence UCSC hg19 assembly (NCBI construction 37.2), using the genome analysis tool, Picard software, dbSNP 132. We also found two separate programs for recognizing somatic mutation cells: MuTect 1.1.5 and VarScan 2.3.6.

### Kidney Transplant Tissue Samples and RNA Sequencing Methods

We collected kidney transplant tissues in three recipients who were subjected to transplanted kidney biopsy due to proteinuria operated at First Affiliated Hospital with Nanjing Medical University between 2016 and 2018. In addition, three normal kidney samples were obtained from patients undergoing radical nephrectomy, and each sample was excised at 5 cm away from tumor tissue. The collected samples were stored at −80°C for the preparation of RNA extraction. And all the samples were analyzed separately.

Total RNA was used as an input material for the RNA sample preparations. Sequencing libraries were generated using NEBNext^®^ UltraTM RNA Library Prep Kit for Illumina^®^ (NEB, United States) following the manufacturer’s recommendations, and index codes were added to attribute sequences to each sample. Briefly, mRNA was purified from total RNA using poly-T oligo–attached magnetic beads. Fragmentation was carried out using divalent cations at elevated temperature in NEBNext First Strand Synthesis Reaction Buffer (5X). First strand cDNA was synthesized using the random hexamer primer and M-MuLV Reverse Transcriptase (RNase H). Second strand cDNA synthesis was subsequently performed using DNA Polymerase I and RNase H. Remaining overhangs were converted into blunt ends *via* exonuclease/polymerase activities. After adenylation of 3′ ends of DNA fragments, NEBNext Adaptor with a hairpin loop structure was ligated to prepare for hybridization. In order to select cDNA fragments of preferentially 250–300 bp in length, the library fragments were purified with an AMPure XP system (Beckman Coulter, Beverly, United States). Then, 3 μl USER Enzyme (NEB, United States was used with size-selected, adaptor-ligated cDNA at 37°C for 15 min followed by 5 min at 95°C before PCR. Then, PCR was performed with Phusion High-Fidelity DNA polymerase, Universal PCR primers, and Index (X) Primer. At last, PCR products were purified (AMPure XP system), and library quality was assessed on the Agilent Bioanalyzer 2100 system.

### Identification and Elucidation of Differentially Expressed Genes (DEGs)

DEGs were screened with ∣log  fold‐change (FC) | >1 and *p*-value < 0.05 between the proteinuria and normal groups, using the limma R package (version 3.13). The Gene Ontology (GO) functional annotation and Kyoto Encyclopedia of Genes and Genomes (KEGG) pathway analysis of the identified DEGs were performed on DAVID 6.8 (https://david.ncifcrf.gov/). *p*-Value<0.05 was considered statistically significant.

### Immunohistochemistry (IHC) Staining Assay

Kidney samples were fixed in 10% neutral formalin and embedded in paraffin. Paraffin sections (3 μm) were deparaffinized, hydrated, and antigen-retrieved. The endogenous peroxidase activity was quenched by 3% H_2_O_2_. Sections were then blocked with 10% normal donkey serum, followed by incubation with anti‐TLR2 (1:100; Abcam, United States) overnight at 4°C. After incubation with biotinylated goat anti‐mouse/rabbit IgG (0.5 μg/ml; Abcam) for 1 h, sections were incubated with ABC reagents for 1 h at room temperature before being subjected to substrate 3-amino-9-ethylcarbazole or 3,3′-diaminobenzidine (Vector Laboratories, Burlingame, CA). Slides were viewed under a Nikon Eclipse 80i microscope equipped with a digital camera (DS-Ri1, Nikon, Shanghai, China).

### Cell Culture and Transfection

Human podocytes cryopreserved at −80°C were resuscitated with 10% FBS-containing RPMI 1640 medium, maintained at 33°C (permissive conditions) and 5% CO2, and cultured at 37°C (non-permissive conditions). After 14 days under non-permissive conditions, the cells revealed an arborized shape. Podocytes digested with 0.25% trypsin for passage after differentiating and passaged according to cell growth once every 2–3 days.

Before transfection, human podocytes were starved with FBS-free RPMI 1640 medium overnight. Then, cell transfection was performed according to the manufacturer’s protocol.

Plasmid purification, restriction digestion, endonuclease digestions, gel electrophoresis, PCR, ligation, and *E. coli* transformations were carried out by Hanheng Biotechnology (Hanheng Biotechnology Co., Ltd., Shanghai, China) (Supplementary Table S1). Briefly, we designed the primer sequences of rs3804099 mutation and TLR2 wild-type, respectively. The digested products were electrophoresed to separate restriction fragments in agarose gel. The digested vector and PCR fragment were ligated and used to transform DH5α competent cells. The two target fragments (wild-type and SNP mutant type) were ligated to the vector pcDNA3. Products were transformed and propagated in DH5α competent cells. Then, they were grown in lysogeny broth (LB) or on LB agar plates and incubated in an incubator at 37°C for 12 h. The monoclonal colonies were selected for amplification and identified by bacterial liquid PCR. Final plasmid constructs were confirmed by DNA sequencing. The concentration of the plasmids was more than 200 ng/μl. The final derived plasmids are referred to as pCDNA3.1 and pCMV-TLR2 (mut) and used for subsequent cell experiments. In our previous study, we have validated that transfection of an empty plasmid is identical to that of the wild-type plasmid (Wang et al., 2020).

### Flow Cytometry Detecting FITC-Annexin V Positive Apoptotic Cells

The cell apoptosis was detected by the FITC-Annexin V Apoptosis Detection Kit (Cat#556547, BD Pharmingen) as described previously. Briefly, the cells with indicated treatment were stained with FITC-Annexin V and propidium iodide (PI). Both early (Annexin V+/PI-) and late (Annexin V+/PI+) apoptotic cells were sorted by fluorescence-activated cell sorting (FACS) (Beckman Coulter Inc., Brea, CA).

### Western Blot Analysis

To extract the total proteins, cells were washed twice with cold PBS and lysed in lysis buffer (0.2% SDS, 1% NP-40, 5 mM EDTA, 1mM PMSF, 10 g/ml leupeptin, and 10 g/ml aprotinin) after treatment. Lysates were centrifuged at 4°C for 20 min at 1,500 g. Protein concentrations were determined by a Bradford assay (Bradford, 1976). Afterward, the proteins were separated by 12% SDS-polyacrylamide gel electrophoresis (PAGE) and transferred onto polyvinylidene difluoride (PVDF) membranes. Membranes were blocked with 5% non-fat milk in TBS (pH 7.4) with 0.1% Tween-20 for 1 h at room temperature and then incubated overnight at 4°C with different primary antibodies. The signals were detected by HRP-conjugated secondary antibodies for 1 h at room temperature on an ECL detection system (Amersham Biosciences). To isolate proteins in the cytoplasm and nucleus, the Nucleus Protein Extraction Kit was used according to the instructions (Boster, Wuhan, China). The primary antibodies were listed as follows: anti‐TLR2 (1:1,000; Abcam, United States), anti‐Desmin (1:1,000; Abcam, United States), anti‐Podocalyxin (1:1,000; Abcam, United States), anti‐Nephrin (1:1,000; Abcam, United States), anti‐GAPDH (1:1,000; CST, United States), anti‐Bcl-2 (1:1,000; CST, United States), anti‐Bax (1:1,000; CST, United States), and anti‐Cleaved Caspase3 (1:1,000; CST, United States). Proteins in the cytoplasm and nucleus were checked by western blot assays and were normalized to GAPDH. The relative intensities of the signals were quantified by densitometry and imaging software (ImageJ, National Institutes of Health, United States).

### Statistical Analysis

Unless otherwise stated, data were expressed as mean ± standard deviation (SD). The minor allele frequency (MAF) and Hardy–Weinberg equilibrium (HWE) were conducted by R package genetics (genetics: Population Genetics, R package version 1.3.8.1.). The linkage disequilibrium (LD) blocks were analyzed using Haploview version 4.2 (Broad Institute, Cambridge, MA, United States). General linear models (GLMs) were used to determine the importance and influence of clinical variables on proteinuria. We used the R Statistics Package SNPassoc (SNP-based whole genome association studies; R package version 1.9-2.) to examine five multiple inheritance models [codominant model 1 (major allele homozygotes versus heterozygotes), codominant model 2 (major allele homozygotes versus minor allele homozygotes), dominant model (major allele homozygotes versus minor allele homozygotes plus heterozygotes), recessive model (major allele homozygotes plus heterozygotes versus minor allele homozygotes), log-additive model (major allele homozygotes versus heterozygotes versus minor allele homozygotes), and overdominant model (heterozygotes versus major allele homozygotes plus minor allele homozygotes)]. All data in our study were analyzed using SPSS software version 13.0 (SPSS Inc., Chicago, IL, United States), and *p* < 0.05 was considered statistically significant.

## Results

### Patient Demographics

A total of 97 patients with proteinuria and 103 control kidney-transplanted patients were recruited in this study. The clinical characteristics of the patients, including weight, recipient age, gender, incidence of PRA before kidney transplant, primary or secondary kidney transplant, type of donor, administration of sirolimus, and the presence of delayed graft function (DGF), were collected and compared, as illustrated in Table 1. The study population age ranged from 28 to 52 years, and the mean age of the patients was 36.45 ± 9.46 years. Of the patients with kidney transplant, 70% were male, while 30% were female. None of the patients presented with PRA before kidney transplant. The patients with living donor formed 12%, and the patients with donor after cardiac death (DCD) formed 88%. Twelve patients were treated with sirolimus.

**TABLE 1:** Basic demographics of kidney transplantation patients in this cohort.

Characteristics	PKT (*n*)	Non-PKT (*n*)
Case number	97	103
Weight (kg; mean ± SD)	60.09 ± 9.21	60.94 ± 9.03
Recipient age (years; mean ± SD)	37.31 ± 9.98	35.65 ± 8.92
Gender (male/female)	72/25	69/34
PRA before renal transplant (%)	0	0
Primary/secondary renal transplant		
Primary renal transplant	97	103
Secondary renal transplant	0	0
Type of donor		
Living donor	15	9
DCD	82	94
Administration of sirolimus (%)	11.34	12.62
DGF (%)	37.11	29.13

Abbreviation: SD, standard deviation; PRA, panel reactive antibody; DCD, donor after cardiac death; DGF, delayed graft function; PKT, proteinuria after kidney transplantation.

### Tagger SNP Selection

**FIGURE 1:**
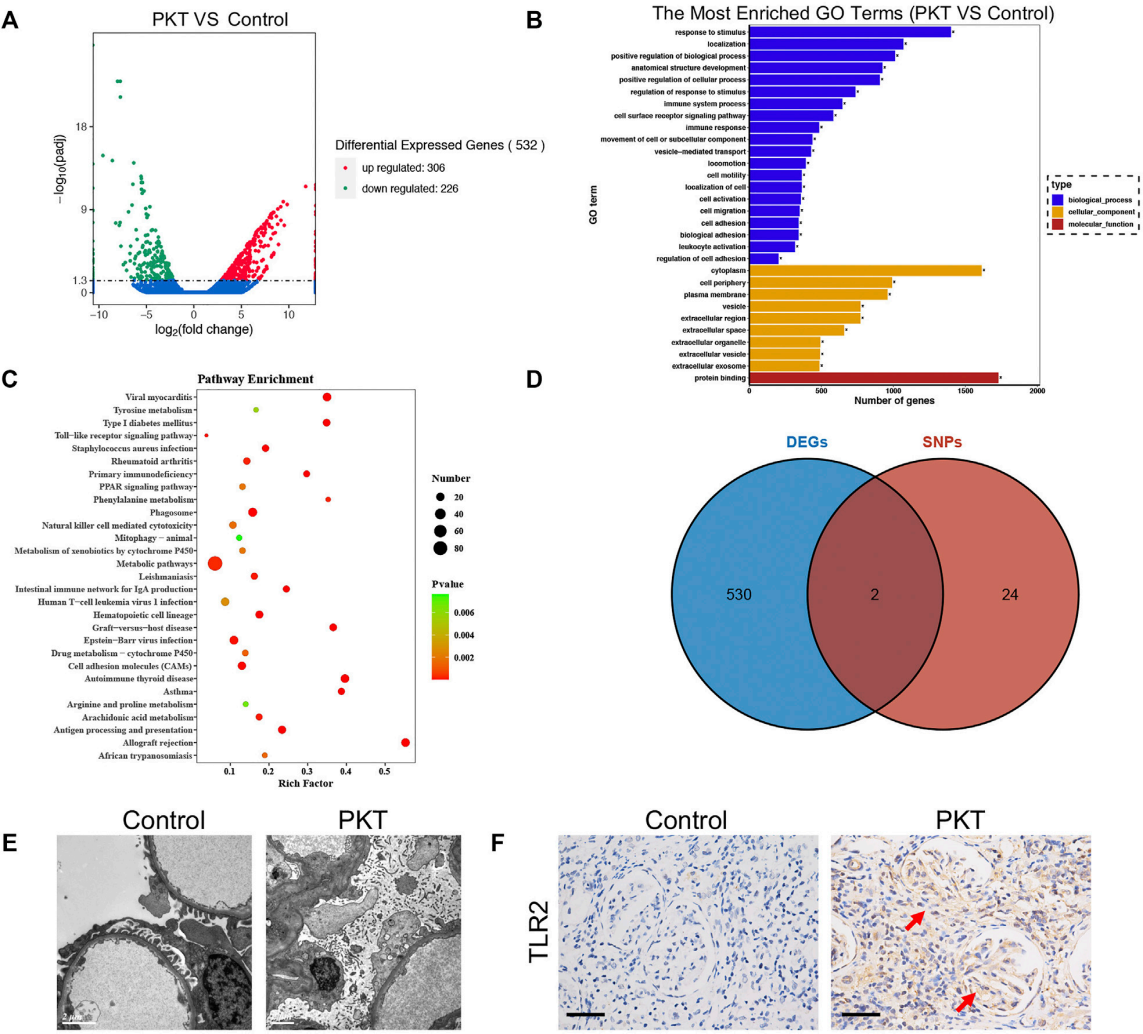
Identification and functional annotation of DEGs and validation of TLR2 expression in tissue. **(A)** Volcano plots of 532 DEGs are shown between the PKT and control groups with 306 upregulated genes and 226 downregulated genes. **(B)** GO enrichment analysis–depicted DEGs are associated with the immune system and inflammatory response. **(C)** KEGG analysis of the DEGs showing the top 20 enriched KEGG terms including toll−like receptor signaling pathway. **(D)** Venn diagram exhibiting two intersected genes including the TLR2 gene between DEGs and SNP mutation–related genes. **(E)** Podocyte foot effacement in podocytes of glomeruli is seen in the PKT group under electron microscopy compared with the control group. **(F)** IHC staining showing TLR2 expression is higher in the PKT group than the control group. Abbreviation: DEGs, differentially expressed genes; PKT: proteinuria after kidney transplantation; GO, Gene Ontology; KEGG: Kyoto Encyclopedia of Genes and Genomes; SNP, single-nucleotide polymorphism; IHC: immunohistochemistry.

**FIGURE 2:**
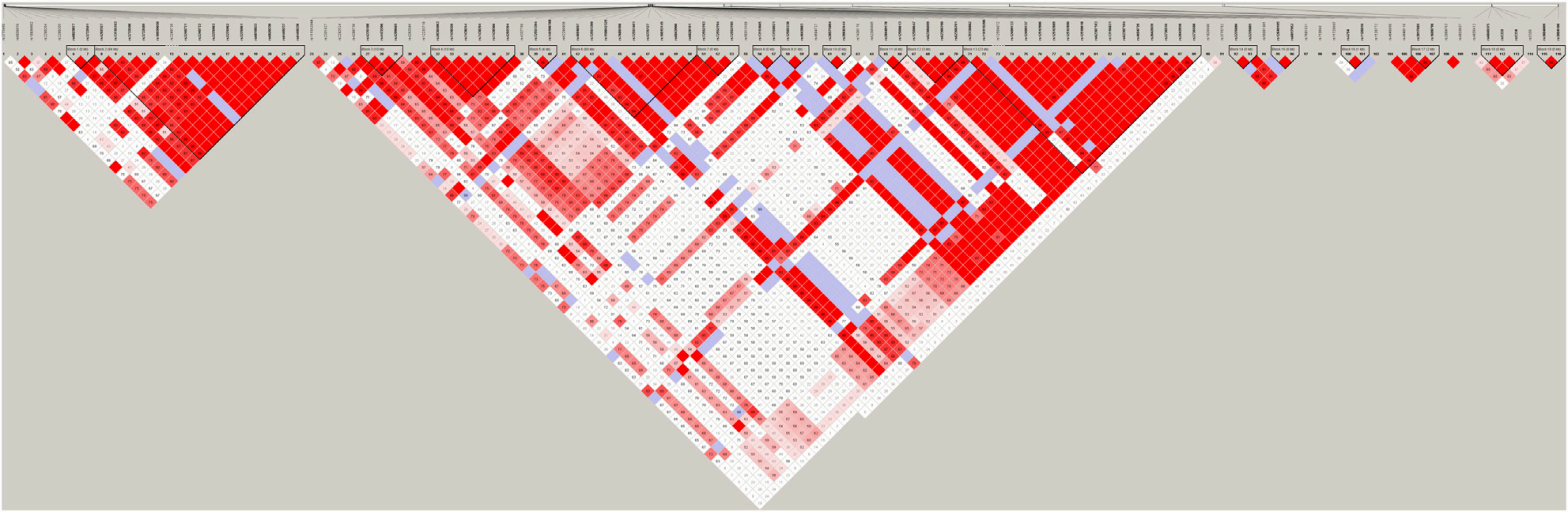
LD results of detected SNPs in the TLR2 gene. Abbreviation: LD, linkage disequilibrium; SNPs, single-nucleotide polymorphisms.

We identified SNPs of 26 genes (Supplementary Table S2) for further analysis using HWE analysis and filter criteria of MAF>0.05. These SNPs were deemed as statistically frequent, whereas the remaining were identified to be rare. As shown in Figure 1A, DEGs between control and PKT (proteinuria after kidney transplantation) groups (*n* = 3) were evaluated by RNA sequencing. Red represents an upregulated gene, and green represents a downregulated gene. By assessing the sequencing results, 532 genes including 306 upregulated and 226 downregulated genes were found differentially expressed between the two groups, based on the screening condition of ∣log_2_ fold-change∣>1 and *p*-value < 0.05. GO enrichment analysis suggested that these DEGs were related to the immune system and inflammatory response, such as cell adhesion (GO:0007155) and regulation of cell adhesion (GO:0030155) (Figure 1B). KEGG pathway enrichment analysis of the DEGs showed the top 20 enriched KEGG terms, including toll−like receptor signaling pathway, natural killer cell mediated cytotoxicity, and mitophagy (Figure 1C). In addition, two genes including TLR2 and SLC2A9 were selected from the intersection between the 532 DEGs and 26 SNP mutated genes (Figure 1D). According to the KEGG pathway analysis (Figure 1C), toll−like receptor signaling pathway was among the top 20 enriched KEGG terms. Besides, previous studies support that TLR2 is closely associated with proteinuria (Motojima et al., 2010; Ururahy et al., 2012; Bergallo et al., 2017). Considering that, we selected TLR2 for further analysis. Furthermore, LD analysis was performed among the SNPs to discover the tagger SNPs (Figure 2). We take the intersection between SNPs selected by LD analysis and frequent SNPs by HWE/MAF analysis. Hence, a couple of tagger SNPs (rs3804099, rs3804100) from the TLR2 gene were used as representative loci for the subsequent statistical analysis.

For further verifying the impact of TLR2 on the development of proteinuria, experiments using human specimens with (PKT group) or without (control group) proteinuria after kidney transplantation were implemented. IHC staining results showed that the expression of TLR2 significantly increased in the PKT group compared with the control group (Figure 1F). Additionally, more podocyte foot effacement in podocytes of glomeruli was observed on electron microscopy in the PKT group than the control group (Figure 1E).

### Confounding Factor Analysis and Multiple Inheritance Model Analysis

Based on various degrees of correlation with proteinuria, confounding factors, including BKV infection, incidence of acute rejection gender, age, weight, ISD protocol, duration after renal transplant, use of rapamycin, and DGF occurrence, were evaluated by GLM analysis. According to the GLM results, duration after kidney transplantation impacted the generation of proteinuria (*p* = 0.042), whereas other factors made no difference to that (*p* > 0.05) (Table 2).

**TABLE 2:** Influence of confounding factors on the outcomes of proteinuria by a general linear model in this cohort.

Confounding factors	*F* value	*p-Value*
BKV infection	0.829	0.407
AR	0.36	0.719
Gender	1.311	0.190
Age	1.741	0.082
Weight	−1.212	0.225
ISD protocol	−1.134	0.257
Duration after kidney transplantation	2.033	**0.042**
RAPA	−0.055	0.956
DGF	0.563	0.573

Abbreviation: ISD, immunosuppressive drug; RAPA, rapamycin; DGF, delayed graft function. The bold values are statistically significant.

A Bonferroni multiple-testing correction was used to adjust the different duration after kidney transplantation of kidney transplant patients (adjusted *p*-value = .005), and multiple inheritance model analyses were performed to narrow down the candidate SNPs. After massive univariate testing, SNP rs3804099 was found significantly associated with proteinuria after kidney transplantation [codominant model: OR (95% CI) = 1.91 (1.01, 3.62), *p* < 0.001; dominant model: OR (95% CI) = 2.38 (1.29, 4.41), *p* = 0.005; recessive model: OR (95% CI) = 7.19 (1.92, 26.97), *p* < 0.001; overdominant model: OR (95% CI) = 1.3 (0.72, 2.35), *p* = 0.380; log-additive model: OR (95% CI) = 2.48 (1.5, 4.09), *p* < 0.001] (Table 3). This result suggested that the risk of proteinuria was strongly correlated with the rs3804099, rs3804100 locus (Supplementary Table S3), compared with other non-significant SNPs (*p* > .05). After searching the literature studies carefully, we found rs3804099 SNP has been reported closely associated with some diseases, including polycystic ovary syndrome (Kuliczkowska-Płaksej et al., 2021) and *Helicobacter pylori* infection and peptic ulcer (Mirkamandar et al., 2018). And rs3804099 polymorphism also has an impact on the susceptibility of cancers (Gao et al., 2019). According to that, we selected rs3804099 for further analysis.

**TABLE 3:** Results of multiple inheritance models in rs3804099 adjusted by the administration of sirolimus in five models.

rs3804099	OR (95% CI)	*p*-Value
Codominant model	1.91 (1.01, 3.62)	**<0.001**
Dominant model	2.38 (1.29, 4.41)	**0.005**
Recessive model	7.19 (1.92, 26.97)	**<0.001**
Overdominant model	1.3 (0.72, 2.35)	0.380
Log-additive model	2.48 (1.5, 4.09)	**<0.001**

Abbreviation: OR, odds ratio; CI, confidence interval. The bold values are statistically significant.

Furthermore, we selected three genotypes and compared differences between the degrees of proteinuria for each of the three groups. The results indicated significant differences (*p* = 0.005) for degrees of proteinuria among the CC, TC, and TT genotypes (Table 4). The analysis results further showed that patients with two T alleles (TT genotypes) showed a significant decreased risk in the development of proteinuria after transplantation.

**TABLE 4:** Distributions and analysis of rs3804099 in patients with proteinuria.

Genotype	Proteinuria degree					*p-*Value
	-	1+	2+	3+	4+	
TC	46	35	13	2	2	**0.005**
TT	54	24	4	1	1	
CC	3	13	2	0	0	

The bold values are statistically significant.

### Rs3804099 Enhanced Expression of TLR2 and Induced Podocyte Injury and Apoptosis

**FIGURE 3:**
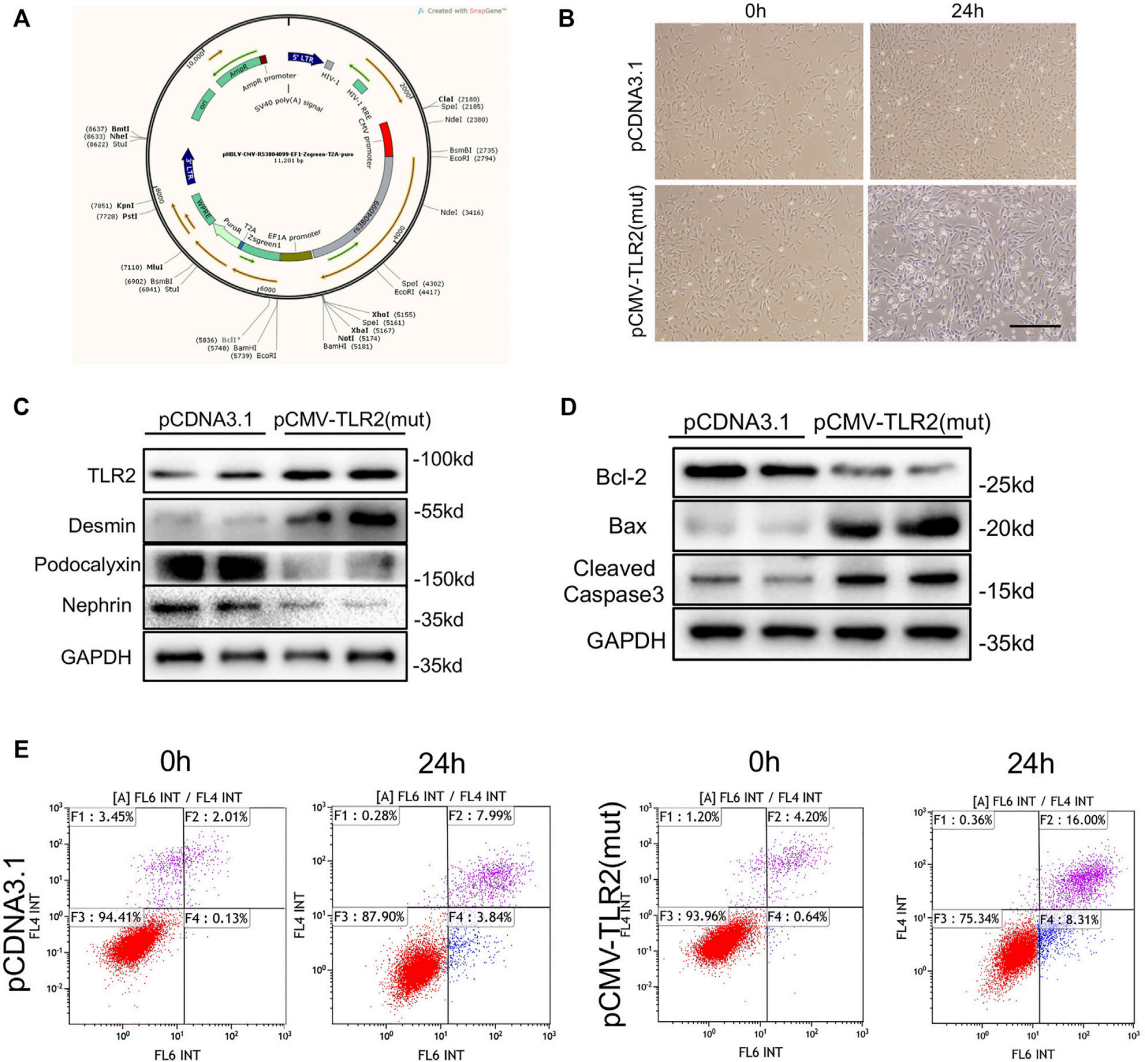
Rs3804099 mutation on the TLR2 gene contributed to cell injury and apoptosis in podocytes. **(A)** Wild-type (pCDNA3.1) and mutated (pCMV-TLR2) plasmids of rs3804099 were constructed for further verification. **(B)** After transfection for 24 h, podocytes in the mutant group showed massive morphological alterations compared with the wild-type group. **(C)** Western blotting assays showed that mutant plasmids transfected in podocytes could significantly reduce podocalyxin and nephrin expression levels but enhance the expression level of desmin, coupled with the increased expression of TLR2. **(D)** The protein level of Bcl-2 was reduced and Bax and cleaved-caspase-3 expressions were increased in the mutant group compared with the wild-type group. **(E)** Flow cytometry results revealed that the cell apoptosis rate was remarkably increased in a time-dependent manner in the mutant group compared with the wild-type group.

To identify the influence of rs3804099 on podocytes, the pCDNA3 plasmids and the pCDNA3.1 plasmids with rs3804099 mutation were constructed (Figure 3A). Then, podocytes transfected with the pCDNA3.1 or pCMV-TLR2 plasmids were assigned to the control or mutant groups, respectively. After transfection for 24 h, podocytes in the mutant group showed massive morphological alterations detected by phase-contrast microscopy (Figure 3B). Using the western blotting assays, we assessed the impact of the rs3804099 site and found the increase of TLR2 expression in the rs3804099 mutant group (Figure 3C).

Nephrin and podocalyxin are the most important components of the slit membrane of podocytes. Western blotting assays showed that the mutant plasmid transfected in podocytes could significantly reduce podocalyxin and nephrin (podocyte marker proteins) levels but enhance the expression of desmin (a marker of podocyte injury) level, coupled with the increased expression of TLR2. Moreover, the protein level of Bcl-2 was reduced and Bax and cleaved-caspase-3 expressions were increased in the mutant group compared with the wild-type group (Figure 3D). Consistently, flow cytometry results revealed that the cell apoptosis rate was remarkably increased in a time-dependent manner in the mutant group compared with the wild-type group (Figure 3E).

## Discussion

The occurrence of proteinuria is one of the evaluation indicators of kidney transplantation damage, and it is accompanied by renal function damage and becomes an independent risk factor for poor prognosis after kidney transplantation. It has been reported that the quantification and duration of proteinuria after kidney transplantation are positively correlated with the risk of renal allograft function damage and even loss of renal allograft. When proteinuria increases by 100 mg, the risk of renal allograft loss greatly increases (Vachek et al., 2020). The current clinical prevention and treatment measures are reasonable dietary adjustment, blood lipid control, hormones, and immunosuppressive agents to reduce the progressive damage of the kidney’s innate cells, but the effect is not satisfactory (Opelz et al., 2006). Therefore, early detection and prediction of proteinuria are particularly important.

Nowadays, with significant improvements in reliability, sequencing chemistry, data interpretation, and costs, next-generation sequencing (NGS) has been increasingly applied in clinical practice, especially diagnostics. Based on the NGS techniques using massively parallel sequencing to decode large areas of the genome, we could potentially identify underlying causes of the disease, predict responses to interventions, and determine individuals at risk. To date, NGS has achieved great advances in infectious diseases (Gu et al., 2019), oncology (Kamps et al., 2017), and reproductive health (Mellis et al., 2018). Besides, Nagano C et al. found the common podocyte-related genes with mutations causing proteinuria were WT1, NPHS1, INF2, TRPC6, and LAMB2, by comprehensive gene screening of patients with diagnosis of nephrotic syndrome or glomerulosclerosis (Nagano et al., 2020). Bedin et al. (2019) reported 4 C-terminal CUBN variants are associated with proteinuria and slightly increased GFR, according to the NGS results from patients with suspected hereditary renal disease and chronic proteinuria. In our study, relying on the NGS technique, the TLR2 gene was detected associated with proteinuria after kidney transplantation. The *in vitro* experiment on podocytes further confirmed our finding.

TLRs are proven to be the human homolog of the *Drosophila* Toll protein. They have similar sequences and structures (Rock et al., 1998). Like *Drosophila* Toll, TLRs are type 1 membrane proteins with a conserved extracellular domain comprising leucine-rich repeats (LRRs) and a cytoplasmic domain related to the IL-1 receptor, termed the “Toll/IL-1 receptor (TIR) homology motif” (Medzhitov et al., 1997). The TLR family is a rapidly expanding family. So far, 11 human TLRs and 13 mouse TLR family members have been identified (O’Neill et al., 2013). Besides located in immunocytes, TLRs are distributed in various parenchymal cells. In the kidney, TLRs were found in both tubular cells and glomerular cells. Therefore, kidney diseases could be affected by the stimulation of TLRs on inflammatory cells or renal cells. Particularly, many experimental and emerging clinical data indicate that TLRs are involved in the pathogenesis of ischemia–reperfusion injury (I/R injury), urinary tract infections (UTIs), acute kidney injury (AKI), diabetic nephropathy, and lupus nephritis (LN).

TLR2 is one of the protein family members of TLRs. It forms TLR2 homodimers, TLR1–TLR2 heterodimers, or TLR2–TLR6 heterodimers to recognize plenty of ligands and triggers inflammatory response depending on the MyD88-signaling pathway. TLR2 can respond to various PAMPs and DAMPs (Shaw et al., 2011). For example, PGN, the main component of the Gram-positive bacteria wall, is considered the important ligand of TLR2. More and more evidence shows that many putative endogenous ligands have been discovered, which belong to the DAMP of TLR2. It is reported that TLR2 responds to endogenous ligands released during cellular stress and injury, such as lipopeptides, high mobility group box 1 (HMGB1), heat shock protein 60 (HSP60), and HSP70 (Asea et al., 2002; de Graaf et al., 2006). Recently, it has been proposed that biglycan, as an extracellular matrix breakdown product, can also activate TLR2. When TLR2 binds to ligands, it thereby activates NF-κB pathways and finally induces proinflammatory cytokines, such as TNF-alpha and IL-6, from immune and non-immune cells. It was reported that TLR2 was expressed in kidney cells such as glomerular endothelial cells and renal tubular cells. It has been reported that proteinuria can cause renal tubular interstitial inflammation, accompanied by activation of the TLR2–MyD88–NF-κB pathway and secretion of proinflammatory cytokines TNF-alpha and IL-6 (Shigeoka et al., 2007). However, the specific mechanism of TLR2 gene acting on proteinuria after kidney transplantation still needs further study.

To sum up, there are many reasons for proteinuria after kidney transplantation, which have cross-effects on the damage of renal allograft function and are critical to the prognosis of the allograft. Monitoring proteinuria levels, and combining with the pathological examination of the transplanted kidney to early diagnose the cause of proteinuria, and corresponding treatment will effectively improve the long-term prognosis of the transplanted kidney. In this study, we indicated that the mutation of rs3804099 in the TLR2 gene was significantly related to the risk of proteinuria after kidney transplantation. The mutation of rs3804099 on the TLR2 gene may be related to podocyte injury and apoptosis. Besides, our data provide insights into the prediction of proteinuria and may imply potential individualized therapy for patients after kidney transplantation. However, limitations still exist in our study. First, three samples from each group were prepared for RNA sequencing, the number of which is too small. Additionally, normal kidney samples for RNA sequencing were excised far away from tumor tissue from patients undergoing radical nephrectomy. Hence, those are not complete normal samples. Besides, most of the patients involved in this study lacked the pathological results of the transplanted kidney biopsy, and the specific causes of proteinuria after kidney transplantation were not analyzed and grouped. Next, we plan to conduct analysis of the etiology of proteinuria to further clarify the roles and mechanisms of TLR2 in proteinuria after kidney transplantation.

## Data Availability

Publicly available datasets were analyzed in this study. The data presented in the study are deposited in the Sequence Read Archive (SRA) database repository, accession number (SRP133091). This data can be found here: [https://www.ncbi.nlm.nih.gov/sra/?term=SRP133091/SRP133091].
