# Supplementary material for: Association Between a TLR2 Gene Polymorphism (rs3804099) and Proteinuria in Kidney Transplantation Recipients
Source: Front Genet. 2022 Feb 21;12:798001. doi: 10.3389/fgene.2021.798001 (PMC8899217; doi:10.3389/fgene.2021.798001)
Supplement: Supplementary file 1 [file Table1.DOCX]

Supplementary Table 1: Detailed information of TLR2 plasmids construction

Vector construction

| **Reagents** | **Companies** | **Lot numbers** |
| --- | --- | --- |
| vector | Hanbio Biotechnology |  |
| DH5α competent cell | TIANGEN | CB101-02 |
| phanta Max-Super-Fidlity DNA polymerase | Vazyme | P505-D1 |
| HB-infusion^TM^ | Hanbio Biotechnology |  |
| Plasmid DNA purification | MACHEREY-NAGEL | 740412 |
| Gel DNA purification | Generay | GK2041 |
| DNA ladder | Generay |  |
| Restriction Endonuclease | Thermo Scientific |  |

Primer design

h-TLR2（MUT）-F:

ctagaggatctatttccggtGaattcGCCACCATGCCACATACTTTGT

h-TLR2（MUT）-R:

GTCACTTAAGCTTGGTACCGAggatccGGACTTTATCGCAGCTCTCAG
