# Supplementary material for: Association Between a TLR2 Gene Polymorphism (rs3804099) and Proteinuria in Kidney Transplantation Recipients
Source: Front Genet. 2022 Feb 21;12:798001. doi: 10.3389/fgene.2021.798001 (PMC8899217; doi:10.3389/fgene.2021.798001)
Supplement: Supplementary file 2 [file Table2.DOCX]

Supplementary Table 2: 26 TLR2-related genes for the SNP analysis.

| Gene names |
| --- |
| TLR2 |
| SLC2A9 |
| SLCO1B3 |
| SLCO1B1 |
| SLC30A8 |
| RUNX2 |
| RUNX1 |
| RPTOR |
| RORA |
| PRKG2 |
| PRKDC |
| PPARG |
| PINLYP |
| PIAS2 |
| PIAS1 |
| PDCD1LG2 |
| PDCD1 |
| OTOP3 |
| NTM |
| NR3C1 |
| NR1I2 |
| NOS3 |
| NKAIN3 |
| NFKB1 |
| MMP10 |
| MMP1 |
