# Supplementary material for: Association Between a TLR2 Gene Polymorphism (rs3804099) and Proteinuria in Kidney Transplantation Recipients
Source: Front Genet. 2022 Feb 21;12:798001. doi: 10.3389/fgene.2021.798001 (PMC8899217; doi:10.3389/fgene.2021.798001)
Supplement: Supplementary file 3 [file Table3.DOCX]

**Supplementary Table 3.** Results of multiple inheritance models in rs3804100 adjusted by the administration of sirolimus in 5 models.

| rs3804100 | OR (95%CI) | *P* value |
| --- | --- | --- |
| Codominant model | 1.76 (0.93, 3.3) | **<0.001** |
| Dominant model | 2.24 (1.22, 4.11) | **0.009** |
| Recessive model | 10.32 (2.21, 48.1) | **<0.001** |
| Overdominant model | 1.21 (0.67, 2.18) | 0.531 |
| log-Additive model | 2.46 (1.49, 4.06) | **<0.001** |

Abbreviations: OR: odds ratio; CI: confidential interval.
